# The Usefulness of C-Reactive Protein to Albumin Ratio in the Prediction of Adverse Cardiovascular Events in Coronary Chronic Total Occlusion Undergoing Percutaneous Coronary Intervention

**DOI:** 10.3389/fcvm.2021.731261

**Published:** 2021-11-12

**Authors:** Lele Cheng, Zixuan Meng, Qi Wang, Zhijie Jian, Pengcheng Fan, Xinxin Feng, Xiangrui Qiao, Jian Yang, Zuyi Yuan, Bolin Li, Yue Wu

**Affiliations:** ^1^Department of Cardiovascular Medicine, The First Affiliated Hospital, Xi'an Jiaotong University, Xi'an, China; ^2^Department of Medical Imaging, The First Affiliated Hospital, Xi'an Jiaotong University, Xi'an, China; ^3^Key Laboratory of Molecular Cardiology, Xi'an, China; ^4^Key Laboratory of Environment and Genes Related to Diseases, Ministry of Education, Xi'an, China; ^5^First Affiliated Hospital of Xinjiang Medical University, Ürümqi, China

**Keywords:** c-reactive protein to albumin ratio, percutaneous coronary intervention, chronic coronary total occlusion, adverse cardiovascular events, prognostic indicator

## Abstract

Inflammation and nutrition as main factors can affect the prognosis of patients with chronic total coronary occlusion (CTO) undergoing percutaneous coronary intervention (PCI). The C-reactive protein to albumin ratio (CAR) can clarify the inflammation and nutrition status, which are highly related to clinical outcomes. This study aims to investigate the association between CAR and adverse cardiovascular events in patients with CTO undergoing PCI. For this study, 664 patients were divided into three groups based on the tertiles of CAR. The primary endpoint was all-cause mortality and the secondary endpoint was major adverse cardiovascular events (MACE). Over a median follow-up of 33.7 months, the primary endpoint occurred in 64 patients (9.6%) and the secondary endpoint occurred in 170 patients (25.6%). The patients with higher CAR represented a worse prognosis with all-cause death and cardiovascular death after the adjustment for the baseline risk factors. Adding the CAR values raised the predictive value for the incidence of the all-cause death and cardiovascular death but not MACE. The capacity of prognosis prediction was improved after the addition of the CAR value to the traditional prediction model.

## Introduction

C-reactive protein, synthesized by hepatocytes, can be used as a biomarker for inflammation, infection and a predictive factor for cardiovascular events (1, 2) Albumin is also synthesized by hepatocytes, has a negative correlation with inflammation, and its levels in plasma can reflect nutrition status which is strongly associated with the prognosis of patients. With the decrease of albumin as well as the increase of C-reactive protein (CRP), it can show impaired immune function, severe inflammation, and worsened prognosis in patients (3, 4).

Among patients with chronic coronary total occlusion (CTO) undergoing percutaneous coronary intervention (PCI), several factors can affect its prognosis, mainly two of which are inflammation and nutrition status. It is known that inflammation plays a key role in coronary heart disease, especially in plaque formation and destabilization, leading to acute coronary syndromes (ACS) or CTO. More than that, it can lead to restenosis in patients with CTO-PCI, causing adverse cardiovascular outcomes and worsening the prognosis (5). In addition, malnutrition is associated with impaired immune function and weak healing ability which are highly related to the mortality of patients with cardiovascular heart disease or other chronic diseases (6, 7). It has been clarified that cytokines and metabolites can be released at malnourished status. Systemic inflammation can activate immune cell transdifferentiation into pro-inflammatory and pro-fibrotic subsets, leading to cardiac hypertrophy and fibrosis (8). For instance, TNF-β as one of the most important inflammatory cytokines increased significantly in patients with malnutrition and its increase resulted in potential negative inotropic effects, worsening ventricular functions, and eventually progressing to heart failure (9). Malnutrition also induced gut microbiota dysbiosis which plays a critical role in cardiovascular disease (CVD), making a difference in the prognosis of patients (10). Moreover, malnutrition usually comes along with the deficiency of micronutrients defined as vitamins and minerals. The lack of Vitamin D is related to the increase of CVD risks resulting from its far-ranging physiological effects (11–14). Supplementation with Vitamin D can prevent CVD by modifying risk factors such as high blood pressure, elevated parathyroid hormone, dyslipidemia, and inflammation (15). Therefore, it is necessary to make an overall consideration of inflammation and nutrition status to better access the prognosis of patients with CTO-PCI.

The emergence of CRP to albumin ratio (CAR) can reflect the inflammation and nutrition status in a better way than either CRP or albumin alone. It has been reported that the elevation of CAR is related to poor prognosis in sepsis, pancreatic cancer, acute pancreatitis, colorectal cancer, and other diseases (16). Studies revealed that CAR is independently associated with no-reflow, acute kidney injury, and coronary thrombus burden in patients with ACS (17–19). C-reactive protein to albumin ratio can be a more practical predictor of the higher presence of left ventricular thrombus formation among survivors of anterior myocardial infarction (20). Nevertheless, the prognostic effect of CAR in CTO remains largely unknown. The presents study aims to investigate whether CAR can be a credible prognostic indicator for patients with CTO undergoing PCI.

## Materials and Methods

### Study Population

**Figure 1:**
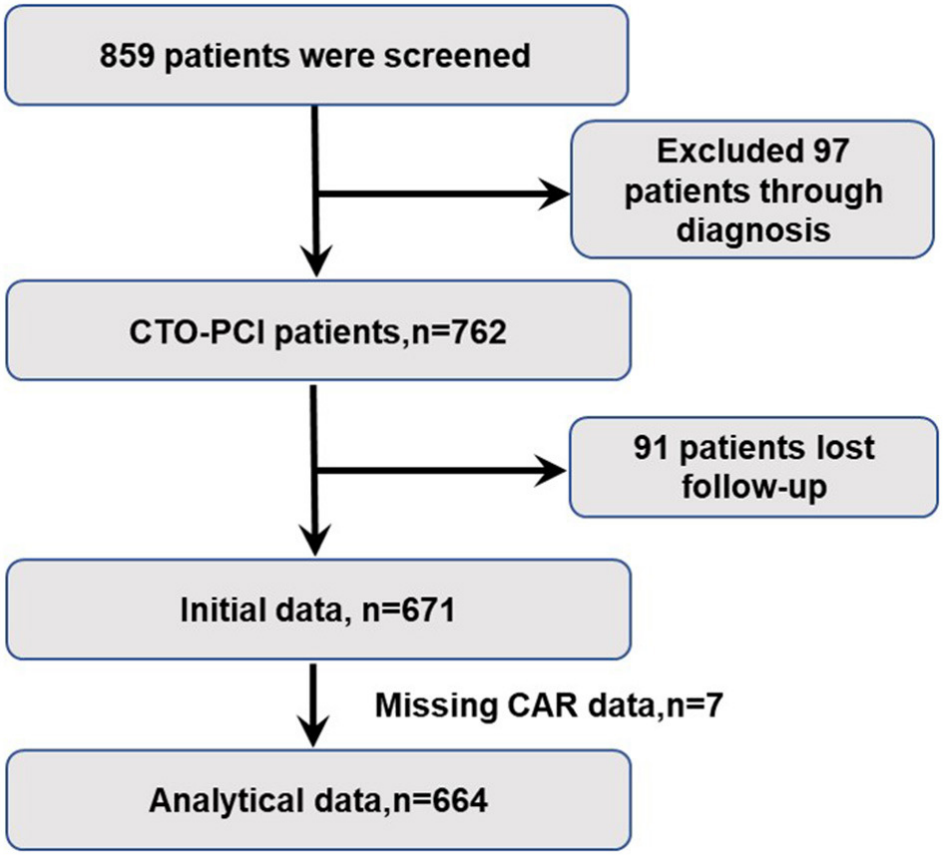
Study design. CTO, chronic total occlusion; PCI, percutaneous coronary intervention.

Between June 2013 to October 2017, 859 patients were screened in the First Affiliated Hospital of Xi'an Jiaotong University and 762 patients received the CTO-PCI strategy. The final follow-up status was ascertained between 2018 and 2019. After the exclusion of 91 patients who were unable to follow-up and 7 patients who missed CAR values, 664 patients were enrolled in the present study (Figure 1).

The patients received standard treatments during the PCI procedure and hospitalization. Coronary total occlusion was defined as total coronary artery occlusion (thrombolysis in myocardial infarction [TIMI] flow grade 0) of more than 3 months in duration (21, 22). The success of the CTO-PCI procedure was defined as the complete revascularization of the lesion with residual stenosis <30% and TIMI flow grade 3 (23). The exclusion criteria were patients with severe renal and hepatobiliary disease diseases, autoimmune diseases, malignant tumors, prior coronary artery bypass grafting, and prior history of surgical treatment within 1 month of this study. The primary endpoint was all-cause mortality and the secondary endpoint was major adverse cardiovascular events (MACE), including all-cause death, non-fatal acute myocardial infarction, revascularization, and stroke. All the participants gave their written informed consent for the enrollment in this study, and the study was approved by the ethics committee approval of the First Affiliated Hospital of Xi'an Jiaotong University.

### Demographic and Laboratory Data

We collected several demographics, cardiovascular risk factors, and laboratory data for all the participants. The demographics included age, gender, and body mass index (BMI). The cardiovascular risk factors included smoking, drinking, hypertension, diabetes mellitus (DM), prior myocardial infarction, and prior stroke, all of which came from the medical records of the participants. Diabetes mellitus was diagnosed according to the criteria by the American Diabetes Association (24). Triglycerides, cholesterol, low-density lipoprotein cholesterol (LDL), high-density lipoprotein cholesterol (HDL), albumin, creatinine, creatine kinase isoenzymes MB (CKMB), and pro-B-type natriuretic peptide (pro-BNP) were assayed using the Cobas Integra automated chemistry analyzer (Roche Cobas Integra 400 Plus, Roche Diagnostics, United States). High-sensitivity CRP was tested using an enzyme-linked immunosorbent assay according to the instructions of the manufacturer (EIA-3954, DRG International Inc., Springfield Township, United States). Blood samples were drawn from the patients within 24 h of admission. All of these laboratory tests were implemented using standard methods. Echocardiographs were performed on admission by experienced cardiologists.

### Statistical Analysis

We divided all the patients into three groups based on the tertiles of CAR (Group 1,0.0022–0.0385; Group 2,0.0323–0.0392; Group 3,0.0394–2.1831), and we used the tertile categories in the following analysis. Continuous variables are presented as the mean ± SD if normally distributed or median (lower quartile, upper quartile) otherwise. The categorical variables are presented as numbers and percentages. Differences in the parameters among groups were analyzed using ANOVA for normally distributed variables, the Kruskal-Wallis test was used for non-normally distributed continuous variables, and the chi-square test was used for categorical variables. Spearman correlation coefficients were calculated to assess the association between continuous non-normally distributed variables.

For the prognostic analysis, a Kaplan-Meier curve was applied to compare the prognosis between the three groups based on tertile of CAR. The prognostic value of CAR was assessed by the univariate and multivariate Cox proportional hazards model. Model 1 was adjusted for age and sex. Model 2 included the same variables as model 1, additionally including lifestyle factors (smoking, alcohol drinks), hypertension, DM, cholesterol level, and stent numbers. Model 3 was additionally adjusted for left ventricular ejection fraction (LVEF), pro-BNP, creatinine, heart rate, revascularization, cholesterol, triglyceride, and thyroid-stimulating hormone (TSH). A Cox proportional hazards regression analysis was performed to estimate the hazard ratios (HRs) and 95% CIs for adverse outcomes among the tertiles of CAR. Survival analyses were performed using the R package “survival” (https://CRAN.R-project.org/package=survival) and “survminer” (https://CRAN.R-project.org/package=survminer).

Aiming to assess whether the accuracy of predicting clinical outcomes improved after the addition of CAR to a baseline model with established risk factors, including age, gender, smoking, alcohol drinks, hypertension, diabetes mellitus, prior myocardial infarction, cholesterol, CKMB, LVEF, and pro-BNP, the C-index, net reclassification improvement (NRI), and integrated discrimination improvement (IDI) were calculated. The IDI and NRI were performed using the R package “PredictABEL” (https://CRAN.R-project.org/package=PredictABEL). Receiver operating characteristic curve analyses were performed using the R package “Proc” (https://CRAN.R-project.org/package=pROC).

Restricted cubic splines were applied using the R package “rms” (https://CRAN.R-project.org/package=rms) to explore the relationship of CAR with the HR of all-cause death, cardiovascular death, and MACE adjusted by potential confounding factors as Model 3 used in Cox regression analysis. The reference value was the CAR_−L_ group.

The *p* < 0.05 was regarded as statistically significant for all statistical tests. All statistical analyses were performed using R version 4.0.2 software (Vienna, Austria).

## Results

### Entire Population Findings

The CAR ranged from 0.0022 to 2.1831. The patients were divided into 3 groups according to the tertile of CAR: CAR_−L_ (*n* = 221), CAR_−M_ (*n* = 222), CAR_−H_ (*n* = 221). The clinical characteristics and medications of the patients are summarized in Table 1. The mean patient age was 65.31 ± 10.00 years, 83.4% of patients were men, 36.0% had diabetes mellitus, and 55.6% had hypertension. Compared with the groups with low CAR values, the patients in the CAR-_H_ group were older with higher hs-CRP, cholesterol, LDL, pro-BNP level, and lower serum albumin. There were no significant differences in other cardiovascular risk factors among the three groups. The success rate of the revascularization was similar. The compliance with medication among the medication use of the patients with CTO after PCI are as follows: aspirin (72.6%), clopidogrel (37.9%), statin (61.1%), ACEI/ARB (28.6%), β-blocker (52.7%), and CCB (21.7%).

**Table 1:** Characteristics of patients according to tertiles by CAR.

	**CAR_**-L**_ (*n =* 221)**	**CAR_**-M**_ (*n =* 222)**	**CAR_**-H**_ (*n =* 221)**	***P*-Value**
CAR	0.018 (0.010, 0.028)	0.036 (0.034, 0.037)	0.081 (0.045, 0.147)	-
Age, years	63.75 ± 9.98	64.97 ± 9.60	67.21 ± 10.13	<0.001
Male, %	184 (83.3)	184 (82.9)	186 (84.2)	0.892
BMI, kg/m^2^	24.30 ± 3.09	25.12 ± 3.60	24.20 ± 3.36	0.791
Smoking, %	114 (51.6)	112 (50.5)	103 (46.6)	0.533
Drunking, %	57 (25.8)	68 (30.6)	47 (21.3)	0.267
Hypertension, %	127 (57.4)	118 (53.2)	124 (56.1)	0.583
Diabetes mellitus, %	79 (35.7)	70 (31.5)	90 (40.7)	0.079
Prior MI, %	70 (31.6)	69 (31.1)	76 (34.4)	0.723
Prior stroke, %	20 (9.0)	22 (9.9)	26 (11.8)	0.635
DBP, mmHg	71.0 (67.0–80.0)	73.0 (68.0, 80.0)	71.0 (65.0, 80.0)	0.500
SBP, mmHg	124.0 (118.0–136.0)	128.0 (116.0, 139.0)	125.0 (114.0, 137.0)	0.726
Heart rate, bmp	70.0 (65.0, 74.0)	70.0 (65.0, 75.0)	70.0 (66.0, 74.0)	0.852
Stent numbers	2.0 (2.0, 3.0)	2.0 (2.0, 3.0)	2.0 (2.0, 3.0)	0.857
Creatinine, mg/dl	67.00 (57.80, 77.00)	67.00 (60.00, 79.70)	70.00 (59.00, 85.64)	0.087
hs-CRP, μg/ml	0.70 (0.40, 1.10)	1.40 (1.40, 1.40)	3.10 (1.80, 5.45)	<0.001
Triglyceride, mmol/l	1.42 (0.97, 2.00)	1.37 (0.98, 2.06)	1.47 (1.11, 2.13)	0.384
Cholesterol, mmol/l	3.54 (2.98, 4.26)	3.63 (3.05, 4.19)	3.77 (3.24, 4.50)	0.008
LDL, mmol/l	2.01 (1.54, 2.55)	2.06 (1.59, 2.60)	2.19 (1.70, 2.85)	0.017
HDL, mmol/l	0.92 (0.81, 1.09)	0.92 (0.78, 1.06)	0.93 (0.81, 1.06)	0.523
Albumin, g/l	40.80 (38.10, 43.60)	39.10 (37.40, 41.00)	37.70 (35.00, 40.40)	<0.001
LVEF, %	60.00 (47.00, 68.00)	61.00 (46.00, 67.00)	58.00 (42.00, 66.00)	0.035
CK-MB, U/l	12.00 (9.35, 15.65)	12.00 (9.20, 15.00)	12.00 (9.00, 16.55)	0.674
pro-BNP, pg/ml	288.65 (91.52, 658.15)	311.90 (146.90, 769.00)	576.90 (211.70, 1765.00)	<0.001
TSH, uIU/ml	2.32 (1.28, 3.37)	2.12 (1.37, 3.20)	1.87 (1.19, 3.13)	0.152
Revascularization, %	150 (67.9)	158 (71.2)	160 (72.4)	0.617
Aspirin, %	164 (74.2)	161 (72.5)	157 (71.0)	0.757
Clopidogrel, %	77 (34.8)	93 (41.9)	82 (37.1)	0.274
Statin, %	135 (61.1)	134 (60.1)	137 (62.0)	0.939
ACEI/ARB, %	55 (24.9)	68 (30.6)	67 (30.3)	0.630
β-blocker, %	116 (52.5)	122 (55.0)	112 (50.7)	0.370
CCB, %	54 (24.4)	45 (20.3)	45 (20.4)	0.445

*CAR, C-reactive protein to albumin ratio; BMI, body mass index; MI, myocardial infarction; DBP, diastolic blood pressure; SBP, systolic blood pressure; LDL, Low-density lipoprotein; HDL, High-density lipoprotein; hsCRP, high sensitivity C-reactive protein; LVEF, left ventricular ejection fraction; CK-MB, creatine kinase isoenzymes MB; pro-BNP, pro-B-type natriuretic peptide; TSH, thyroid stimulating hormone; ACEI, angiotensin-converting enzyme inhibition; ARB, angiotensin receptor blocker. Compliance with medication is indicated*.

### Associations of Conventional Cardiovascular Risk Factors and CAR

The associations between conventional cardiovascular risk factors and CAR are shown in Supplementary Table 1. C-reactive protein to albumin ratio is predominantly associated with hs-CRP (*p* < 0.001), serum albumin (*p* < 0.001), and cholesterol (*p* = 0.046). In contrast, other cardiovascular risk factors including age, LDL, HDL, triglycerides, creatinine, pro-BNP, BMI, LVEF, and TSH showed no associations with CAR.

### Clinical Outcomes

The median duration of follow-up was 33.7 months (interquartile range, 22.5 to 48.9 months) in the whole population. The analyses of the primary and secondary endpoints are provided in Table 2. During the follow-up, the primary endpoint occurred in 64 patients (9.6%) while the secondary endpoint occurred in 170 patients (25.6%).

**Table 2:** Cox proportional hazards analyses of clinical outcomes.

	**CAR_**-L**_** **(*n =* 221)**	**CAR_**-M**_ (*n =* 222)** **HR (95% CI)** ***p*-Value (vs. CAR_**-L**_)**	**CAR_**-H**_ (*n =* 221)** **HR (95% CI)** ***p*-Value (vs. CAR_**-L**_)**	***P* Trend**
**All-cause death**	(*n* = 13)	(*n* = 15)	(*n* = 36)	
Unadjusted	Reference	1.14 (0.54–2.39) *p* = 0.733	2.96 (1.55–5.52) *p* = 0.001	<0.001
Model 1	Reference	1.08 (0.51–2.28) *p* = 0.834	2.66 (1.40–5.05) *p* = 0.003	0.001
Model 2	Reference	1.01 (0.48–2.14) *p* = 0.979	2.61 (1.36–5.02) *p* = 0.004	0.001
Model 3	Reference	0.81 (0.35–1.91) *p* = 0.637	2.26 (1.06–4.79) *p* = 0.034	0.019
**Cardiovascular death**	(*n* = 6)	(*n* = 5)	(*n* = 22)	
Unadjusted	Reference	0.82 (0.25–2.69) *p* = 0.746	3.87 (1.57–9.54) *p* = 0.003	0.001
Model 1	Reference	0.78 (0.24–2.55) *p* = 0.676	3.64 (1.46–9.04) *p* = 0.005	0.001
Model 2	Reference	0.72 (0.22–2.40) *p* = 0.596	3.80 (1.50–9.67) *p* = 0.005	0.001
Model 3	Reference	0.46 (0.12–1.76) *p* = 0.257	3.02 (1.11–8.23) *p* = 0.030	0.012
**MACE**	(*n* = 34)	(*n* = 48)	(*n* = 55)	
Unadjusted	Reference	1.50 (0.92–2.29) *p* = 0.111	1.84 (1.19–2.87) *P* = 0.006	0.006
Model 1	Reference	1.43 (0.91–2.26) *p* = 0.123	1.79 (1.15–2.79) *p* = 0.010	0.009
Model 2	Reference	1.41 (0.89–2.24) *p* = 0.140	1.77 (1.13–2.76) *p* = 0.012	0.012
Model 3	Reference	1.31 (0.80–2.13) *p* = 0.286	1.71 (1.06–2.76) *p* = 0.027	0.026

*Model 1 was adjusted for age, sex. Model 2 included the same variables as model 1+including lifestyle factors (smoking, alcohol drinks), hypertension, diabetes mellitus and sent numbers. Model 3 includes the same variables as model 2+ LVEF, pro-BNP, creatinine, heart rate, revascularization, cholesterol, triglyceride, and TSH. CI, confidence interval; HR, hazard ratio; CAR, C-reactive protein to albumin ratio; BMI, body mass index; MI, myocardial infarction; DBP, diastolic blood pressure; SBP, systolic blood pressure; LDL, Low-density lipoprotein; HDL, High-density lipoprotein; hsCRP, high sensitivity C-reactive protein; LVEF, left ventricular ejection fraction; CK-MB, creatine kinase isoenzymes MB; pro-BNP, pro-B-type natriuretic peptide; TSH, thyroid stimulating hormone; ACEI, angiotensin-converting enzyme inhibition; ARB, angiotensin receptor blocker*.

**Figure 2:**
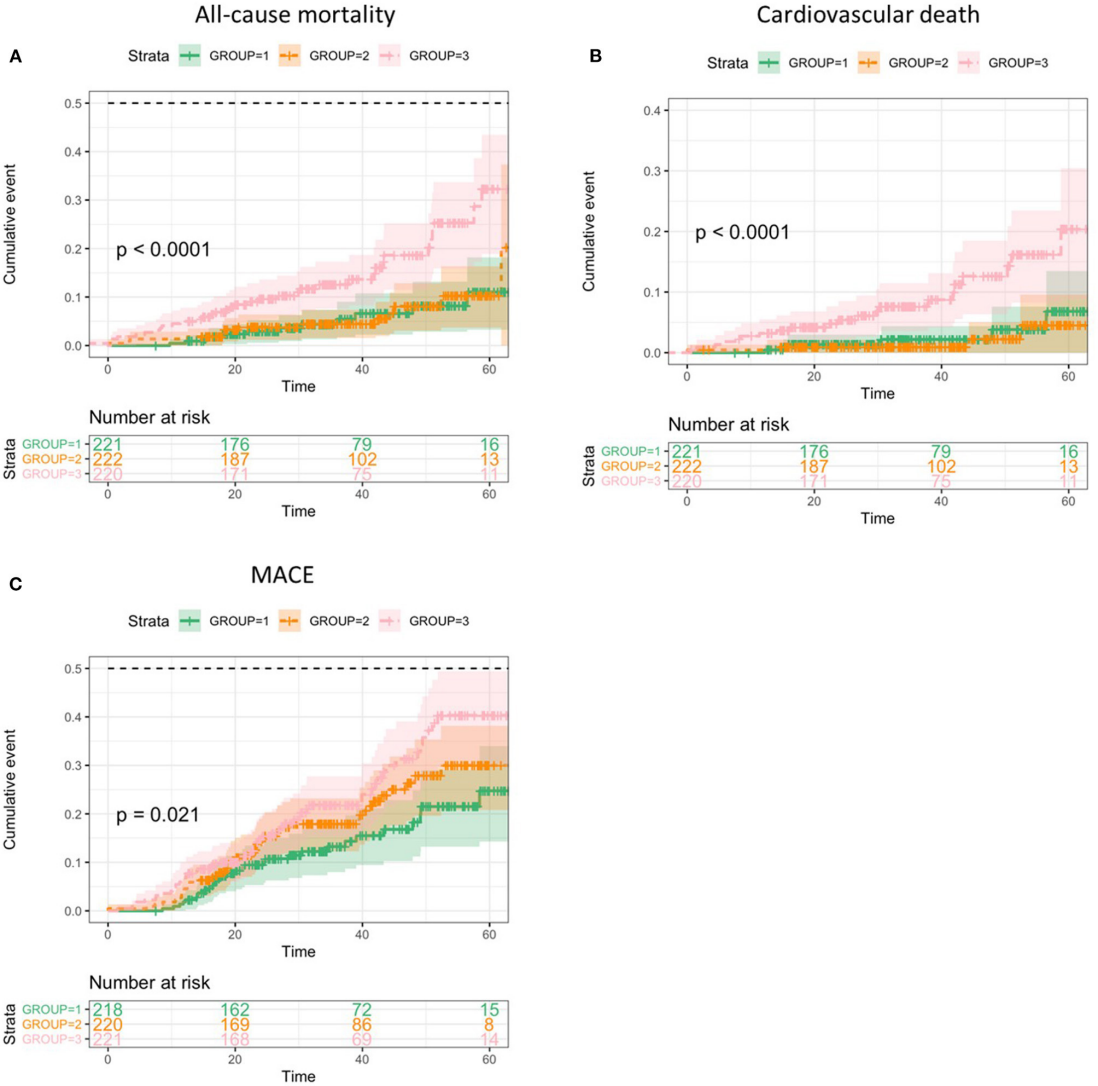
Kaplan-Meier analyses of clinical outcomes categorized by C-reactive protein to albumin ratio (CAR) values. **(A)**, All-cause death. **(B)**, Cardiovascular death. **(C)**, Major adverse cardiovascular events (MACE).

As demonstrated in the Kaplan-Meier plots, the patients with higher CAR represented worse prognosis than the other two groups with all-cause death (log-rank test, *p* < 0.0001), cardiovascular death (log-rank test, *p* < 0.0001), and MACE (log-rank test, *p* = 0.021) (Figure 2).

In the univariate Cox proportional hazards analysis, the patients in the CAR_−H_ group had increased risk of all-cause death (HR:2.96; 95% CI:1.55–5.52; *p* = 0.001, *p* for trend < 0.001), cardiovascular death (HR:3.87; 95% CI:1.57–9.54; *p* = 0.003, *p* for trend = 0.001), and MACE (HR:1.84; 95% CI:1.19–2.87; *p* = 0.006, *p* for trend = 0.006) as compared with the CAR_−L_ group. In the multivariate analysis, the CAR_−H_ group remained associated with increased risk for all-cause death, cardiovascular death, and MACE after the adjustment for models 1, 2, and 3 (Table 2). Additionally, the results of the subgroup analyses for the primary endpoint are provided in Table 3. The results indicated that patients with a high level of CAR implied a worse prognosis in subgroups such as age ≥ 65 years, male, without diabetes mellitus, baseline LDL < 2.46 mmol/L, HDL ≤ 1.04 mmol/L, and triglycerides < 1.68 mmol/L. No significant interaction between the subgroup factors and the effect of CAR was observed (Table 3). Then we chose the patients who have the risk characteristics including age ≥65 years, male, without diabetes mellitus, and baseline LDL < 2.46 mmol/L and found that these patients with high CAR had a 15.23-fold increased risk of having primary endpoint (HR:15.23; 95% CI:1.71–135.95, *p* = 0.015) as compared with those with low CAR values.

**Table 3:** CAR level and risk of all-cause death of CTO-PCI patients, stratified by various baseline characteristics.

**Characteristics**	**CAR_**-L**_**	**CAR_**-M**_**	**CAR_**-H**_**	**Interaction *P-*Value**
**Age**				
<65 y	1 (Reference)	1.31 (0.35-4.89), *p =* 0.687	1.87 (0.55–6.41), *p =* 0.317	0.244
≥65 y	1 (Reference)	1.03 (0.42–2.54), *p =* 0.950	3.40 (1.58–7.28), *p =* 0.002	
**Sex**				
Male	1 (Reference)	1.28 (0.57–2.89), *p =* 0.545	2.86 (1.42–5.77), *p =* 0.003	0.608
Female	1 (Reference)	0.83 (0.12–5.90), *p =* 0.853	4.25 (0.88–20.63), *p =* 0.072	
**Smoking**				
Yes	1 (Reference)	1.35 (0.53–3.44), *p =* 0.524	2.43 (1.03–5.76), *p =* 0.044	0.417
No	1 (Reference)	0.88 (0.25–3.03), *p =* 0.835	3.70 (1.39–9.87), *p =* 0.009	
**Diabetes mellitus**				
Yes	1 (Reference)	0.89 (0.29–2.78), *p =* 0.843	1.92 (0.74–4.99), *p =* 0.183	0.466
No	1 (Reference)	1.52 (0.54–4.28), *p =* 0.431	4.46 (1.79–11.10), *p =* 0.001	
**Hypertension**				
Yes	1 (Reference)	1.53 (0.56–4.16), *p =* 0.409	2.53 (1.04–6.17), *p =* 0.042	0.388
No	1 (Reference)	0.91 (0.29–2.82), *p =* 0.871	3.40 (1.36–8.52), *p =* 0.009	
**LDL**				
≥2.46 mmol/l	1 (Reference)	1.09 (0.26–4.53), *p =* 0.903	1.98 (0.59–6.59), *p =* 0.266	0.074
<2.46 mmol/l	1 (Reference)	1.09 (0.42–2.83), *p =* 0.859	3.88 (1.74–8.63), *p =* 0.001	
**HDL**				
>1.04 mmol/l	1 (Reference)	0.69 (0.17–2.91), *p =* 0.616	2.42 (0.81–7.24), *p =* 0.115	0.942
< =1.04 mmol/l	1 (Reference)	1.14 (0.45–2.90), *p =* 0.780	3.07 (1.37–6.88), *p =* 0.006	
**TG**				
≥1.68 mmol/l	1 (Reference)	0.87 (0.23–3.25), *p =* 0.837	1.44 (0.47–4.42), *p =* 0.522	0.116
<1.68 mmol/l	1 (Reference)	1.06 (0.41–2.77), *p =* 0.902	3.92 (1.73–8.91), *p =* 0.001	

*CAR, C-reactive protein to albumin ratio; BMI, body mass index; MI, myocardial infarction; DBP, diastolic blood pressure; SBP, systolic blood pressure; LDL, Low-density lipoprotein; HDL, High-density lipoprotein; hsCRP, high sensitivity C-reactive protein; LVEF, left ventricular ejection fraction; CK-MB, creatine kinase isoenzymes MB; pro-BNP, pro-B-type natriuretic peptide; TSH, thyroid stimulating hormone; ACEI, angiotensin-converting enzyme inhibition; ARB, angiotensin receptor blocker*.

The C-index of established risk factors including age, sex, smoking, alcohol drinks, hypertension, diabetes mellitus, prior myocardial infarction, cholesterol, CKMB, LVEF, and pro-BNP, for all-cause death (0.767, *p* < 0.001), cardiovascular death (0.812, *p* < 0.001), and MACE (0.587, *p* = 0.003) were revealed. As expected, the C-index was elevated with the addition of CAR,0.776 for all-cause death (*p* < 0.001),0.833 for cardiovascular death (*p* < 0.001), and 0.603 for MACE (*p* < 0.001) (Table 4). The CAR had incremental values for predicting the incidence of the all-cause death (0.451, *p* = 0.001 and 0.013, *p* = 0.049, NRI and IDI, respectively) and cardiovascular death (0.386, *p* = 0.006 and 0.010, *p* = 0.042, NRI and IDI, respectively). Whereas, the NRI and IDI for MACE were not significant after adding CAR (0.182, *p* = 0.129 and 0.002, *p* = 0.443, NRI and IDI, respectively).

**Table 4:** Discrimination of each predictive model for outcomes using C-index, net reclassification improvement (NRI), and integrated discrimination improvement (IDI).

**Predictive Models**	**C-index**	***P*-Value**	**NRI**	***P-*Value**	**IDI**	***P*-Value**
**All-cause death**						
Established risk factors	0.767	<0.001	Reference		Reference	
+CAR	0.776	<0.001	0.451	0.001	0.013	0.049
**Cardiovascular**						
**death**						
Established risk factors	0.812	<0.001	Reference		Reference	
+CAR	0.833	<0.001	0.386	0.006	0.010	0.042
**MACE**						
Established risk factors	0.587	0.003	Reference		Reference	
+CAR	0.603	<0.001	0.182	0.129	0.002	0.443

*Established risk factors included age, sex, smoking, alcohol drinks, hypertension, diabetes mellitus, prior myocardial infarction, cholesterol, CKMB, LVEF, pro-BNP. AUC, area under curve; IDI, integrated discrimination improvement; NRI, net reclassification improvement; CAR, C-reactive protein to albumin ratio; BMI, body mass index; MI, myocardial infarction; DBP, diastolic blood pressure; SBP, systolic blood pressure; LDL, Low-density lipoprotein; HDL, High-density lipoprotein; hsCRP, high sensitivity C-reactive protein; LVEF, left ventricular ejection fraction; CK-MB, creatine kinase isoenzymes MB; pro-BNP, pro-B-type natriuretic peptide; TSH, thyroid stimulating hormone; ACEI, angiotensin-converting enzyme inhibition; ARB, angiotensin receptor blocker*.

**Figure 3:**
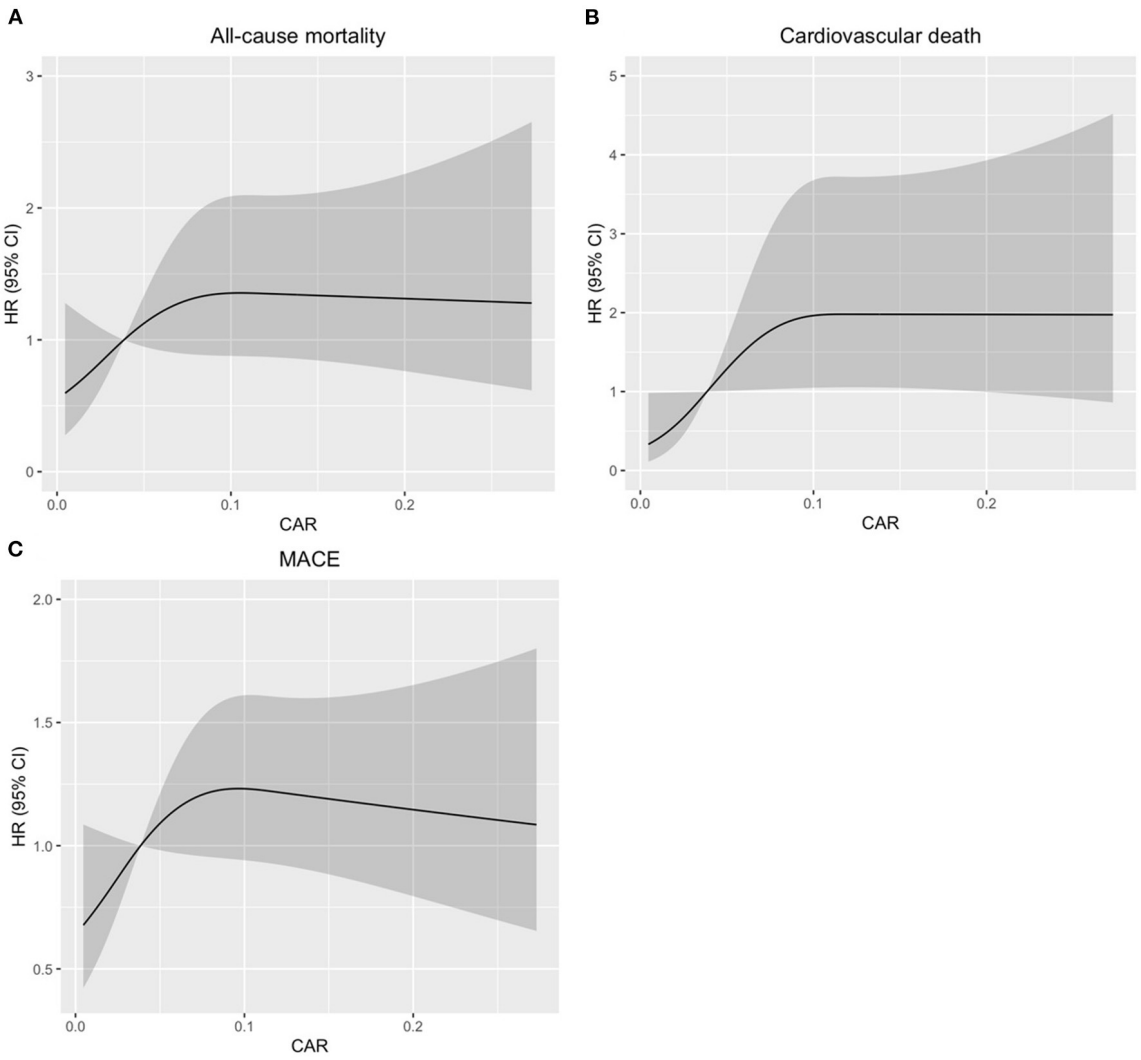
Restricted spline curves for the associations between CAR and all-cause mortality, cardiovascular death, or MACE in patients with coronary total occlusion (CTO) undergoing percutaneous coronary intervention (PCI). Black lines represent the hazard ratio (HR), gray areas represent the 95% CIs. **(A)**, Association between CAR and all-cause mortality. **(B)**, Association between CAR and cardiovascular death. **(C)**, Association between CAR and MACE. The HR (95% CI) were all adjusted according to Model 3 in the Cox analysis.

Further, restricted cubic splines were applied to explore the association of CAR, which was treated as a continuous variable, with the HR of all-cause death, cardiovascular death, and MACE after adjusting as model 3 used in Cox regression analysis. The HR of all-cause death increased sharply until the CAR reached ~0.1. Similar relationships between CAR and cardiovascular death and CAR and MACE were found in the patients with CTO-PCI (Figure 3).

## Discussion

As one of the most prevalent diseases in both developed and developing countries, coronary artery disease (CAD) is still the dominant cause of mortality, especially in elder people (25, 26). With the morbidity of CAD increasing annually, the economic burden gets heavier (27) and CTO, represented by the narrowing of the coronary artery and completely interrupting coronary flow for more than 3 months, is a special form of CAD. Recent research revealed that about 50% of patients with significant CAD on angiography have at least one CTO lesion (21, 28). Although CTO-PCI is regarded as a valid procedure for the technical advancement of angioplasty equipment, it is still full of uncertainties regarding clinical prognosis, mainly due to its complex procedures and high dependence on both the health status of patients and the experience of operators (29).

Inflammation plays a vital role in disease progression and the prognosis of patients, it can not only accelerate the formation of plaque lesions but also promote the progression of complex re-occlusion lesions in elderly patients with very long stent implantations (5, 30). C-reactive protein acts as a biomarker of inflammation and a hallmark of acute-phase response, which can strongly reflect the inflammation status of patients. Previous studies have revealed its pathological role in vascular diseases and the correlation between CRP and the risk of cardiovascular events. C-reactive protein concentration is log linearly related to the risk of CAD (31–33). A moderately elevated CRP is proved to be an independent risk factor for CAD in healthy populations (34–36). Serum albumin levels are negatively related to inflammatory reactions, low serum albumin level is associated with the increased risk of several CVDs in abundant prospective studies (37–40). Up to 55% of elders with STsegment elevation myocardial infractions were malnourished. In addition, malnutrition is quite common in patients with CTO-PCI (41). To evaluate the health status of patients after processing PCI, it is essential to find an effective predictor for the prognosis of patients with CTO-PCI. C-reactive protein to albumin ratio combines these two crucial biomarkers, showing a strong point to clarify the inflammation and nutrition status, both of which are highly related to the disease progression and clinical outcomes of patients. The present study revealed that patients with CTO-PCI with higher CAR values had increased risk for all-cause death, cardiovascular death, and MACE as compared with the traditional prediction model. The addition of CAR remarkably enhanced the ability to predict the risk of adverse cardiovascular events in patients with CTO-PCI. Hence, CAR can be a valid predictor for adverse events in patients with CTO after PCI. To our knowledge, this is the first study to uncover the association between CAR and CTO and the addition of CAR to a clinical prediction model significantly improved the risk prediction for adverse cardiovascular events.

The specific mechanisms of inflammation and malnutrition influencing the prognosis of patients are still unknown. One possibility is that inflammation can restrain the immune system and retard the recovery progression by suppressing the protein synthesis, promoting protein degradation, and lowering plasma albumin. The inflammation cytokines activated by CRP can affect multiple organ functions, worsening the prognosis of patients. C-reactive protein to albumin ratio can also bind to LDL and contribute to the development of atherosclerotic plaques, leading to the incidence of restenosis (42). Serum albumin has anti-inflammatory, anticoagulant, and antiplatelet aggregation activity effects, and low serum albumin levels can reduce serum anti-oxidant activity and impair endothelium-derived relaxing factor-like activities (43, 44). Additionally, albumin can bind to homocysteine to lower the serum levels of homocysteine, which is a risk factor for atherosclerosis (45). Another possibility is that serum albumin can reflect the nutrition status of patients; lower serum albumin is often an indicator of malnutrition. In the state of malnutrition, immune system activity is impaired, leading to multiple complications and a worse prognosis (46). Whereupon, inflammation and malnutrition together form a vicious circle.

It is noteworthy that high CAR had better predictive value in patients of age ≥ 65 years, male, without diabetes mellitus, baseline LDL < 2.46 mmol/L, HDL ≤ 1.04 mmol/L, and triglycerides < 1.68 mmol/L. We deduced that the possible reasons are as follows. Firstly, previous studies based on the population demonstrated that serum albumin levels decreased with aging, which might result from elders suffering worse immunity function, being more vulnerable to malnutrition and chronic inflammation status (46), leading to higher CAR values. Secondly, albumin can be taken as an efficient antioxidant, protecting the organisms from oxidative attack (47, 48). Glucose and free radicals can impair the antioxidant properties of serum albumin (49). That is to say, hyperglycemia may affect the predictive values of CAR. The underlying mechanisms still need to be studied further.

The current study has several limitations that should be considered. Firstly, the clinical data comes from a single-center, small-scale observational cohort study, and some confounding factors may affect the outcomes. Further, multi-center and larger-scale surveys are required to confirm the results from this study and to elucidate the precise mechanisms. Secondly, we only measured the CAR values at admission, without assessing the dynamic changes during the medication. Thirdly, we evaluated CRP as a prognostic biomarker for CVD but lacks data on other inflammatory markers. Moreover, the study only included patients with CTO undergoing PCI, which suggests that the study results may not be extended to all CAD patients.

In summary, our study reveals that the risk of all-cause death, cardiovascular death, and MACE was increased with high CAR values in patients with CTO undergoing PCI. After adding CAR to the traditional prediction model, the capacity of prognosis prediction was improved in these patients. Therefore, CAR can be an indicator to predict the prognosis of patients with CTO undergoing PCI and to better stratify these patients.

## Data Availability Statement

The datasets analyzed during the current study are available from the corresponding author on reasonable request.

## Ethics Statement

The studies involving human participants were reviewed and approved by the Ethics Committee approval of the First Affiliated Hospital of Xi'an Jiaotong University. The patients/participants provided their written informed consent to participate in this study.

## Author Contributions

LC and BL: conceptualization and writing. YW: review and editing. QW, XQ, and BL: data curation. LC and ZM: formal analysis. ZY and YW: funding acquisition. LC, ZM, PF, and XF: investigation. BL: methodology. JY: project administration. ZY: resources. QW: software. ZY, YW, and BL: validation. LC and ZJ: visualization. ZM: writing—original draft. All authors contributed to the article and approved the submitted version.

## Funding

This work was supported by the National Key R&D Program of China (2019YFA0802300) and the National Natural Science Foundation of China (81822005, 91639301, 81500219).

## Conflict of Interest

The authors declare that the research was conducted in the absence of any commercial or financial relationships that could be construed as a potential conflict of interest.

## Publisher's Note

All claims expressed in this article are solely those of the authors and do not necessarily represent those of their affiliated organizations, or those of the publisher, the editors and the reviewers. Any product that may be evaluated in this article, or claim that may be made by its manufacturer, is not guaranteed or endorsed by the publisher.

## Abbreviations

ACEI: angiotensin-converting enzyme inhibitors
ACS: acute coronary syndrome
ARB: angiotensin receptor blocker
BMI: body mass index
CAD: coronary artery disease
CAR: C-reactive protein to albumin ratio
CCB: calcium channel blockers
CIs: confidence intervals
CKMB: creatine kinase isoenzymes MB
CRP: C-reactive protein
CTO: chronic coronary total occlusion
CVD: cardiovascular disease
DM: diabetes mellitus
HbA1c: hemoglobin A1c
HDL: high-density lipoprotein cholesterol
HRs: hazard ratios
IDI: integrated discrimination improvement
LDL: low-density lipoprotein cholesterol
LVEF: left ventricular ejection fraction
MACE: major adverse cardiovascular events
NRI: net reclassification improvement
pro-BNP: pro-B-type natriuretic peptide
PCI: percutaneous coronary intervention
ROC: receiver operating characteristic
TIMI: thrombolysis in myocardial infarction
TSH: thyroid-stimulating hormone.
