# Supplementary material for: The Usefulness of C-Reactive Protein to Albumin Ratio in the Prediction of Adverse Cardiovascular Events in Coronary Chronic Total Occlusion Undergoing Percutaneous Coronary Intervention
Source: Front Cardiovasc Med. 2021 Nov 12;8:731261. doi: 10.3389/fcvm.2021.731261 (PMC8636141; doi:10.3389/fcvm.2021.731261)
Supplement: Supplementary file 1 [file Table_1.DOCX]

**Table S1. Correlations of CAR with other factors.**

|  | Coefficient | P value |
| --- | --- | --- |
| Age | 0.054 | 0.163 |
| LDL | 0.063 | 0.110 |
| HDL | 0.005 | 0.895 |
| Cholesterol | 0.078 | 0.046 |
| Triglycerides | 0.011 | 0.774 |
| Creatinine | 0.028 | 0.466 |
| pro-BNP | 0.016 | 0.679 |
| BMI | 0.028 | 0.515 |
| LVEF | -0.030 | 0.447 |
| hs-CRP | 0.791 | <0.001 |
| Albumin  TSH | -0.316  -0.005 | <0.001  0.899 |

CAR C-reactive protein to albumin ratio; BMI body mass index; LDL low-density lipoprotein; HDL high-density lipoprotein; hs-CRP high sensitivity C-reactive protein; LVEF left ventricular ejection fraction; pro-BNP pro-B-type natriuretic peptide; TSH thyroid stimulating hormone.
